# Genomic analysis of hypoxia and mitophagy related genes with prognosis and characterization of the immune microenvironment in LUAD

**DOI:** 10.7150/jca.91762

**Published:** 2024-01-16

**Authors:** Jinghao Liu, Hua Huang, Yueting Han, Yu Hua, Boshi Li, Hongyu Liu, Jun Chen

**Affiliations:** 1 Department of Lung Cancer Surgery, Tianjin Medical University General Hospital, Tianjin, China.; 2 Department of Clinical Laboratory, Tianjin Medical University Cancer Institute and Hospital, National Clinical Research Center for Cancer, Tianjin's Clinical Research Center for Cancer, Key Laboratory of Cancer Prevention and Therapy, Tianjin, China.; 3 Tianjin Key Laboratory of Lung Cancer Metastasis and Tumor Microenvironment, Tianjin Lung Cancer Institute, Tianjin Medical University General Hospital, Tianjin, China.

**Keywords:** LUAD, hypoxia, mitophagy, genomic analysis, prognosis, immune microenvironment

## Abstract

**Background:** Lung adenocarcinoma (LUAD) stands as a prominent subtype within the realm of non-small cell lung cancer and constitutes a primary contributor to cancer-related mortality on a global scale. Notably, hypoxia, a prevalent attribute within solid tumor environments, and mitophagy, a selective manifestation of autophagy dedicated to the removal of damaged mitochondria, have risen to prominence as pivotal factors influencing the initiation and advancement of tumorigenesis.

**Methods:** This investigation harnessed publicly accessible genomic datasets encompassing LUAD patients to delineate genes linked to hypoxia and mitophagy, termed hereafter as hypoxia and mitophagy-related genes (HMRGs). Large-scale repositories furnished both gene expression profiles and clinical particulars. The expression profiles of HMRGs were meticulously scrutinized across 1,093 LUAD specimens, leveraging resources such as The Cancer Genome Atlas and Gene Expression Omnibus datasets. A methodical exploration of HMRG patterns within LUAD led to the discernment of two distinct molecular subtypes. Moreover, a discernible correlation emerged between the subtypes and their respective clinical attributes. A risk scoring system was formulated to prognosticate overall survival (OS) and therapeutic responsiveness in LUAD patients. Subsequently, the reliability of this scoring system was authenticated, and a nomogram was adopted to refine the clinical utility range of the risk score. The proliferation and migration impacts of KRT8 on LUAD cells were evaluated through cck8 assays, edu assays, and transwell assays, the results were further validated in vivo.

**Results:** Elevated risk scores were indicative of unfavorable OS probabilities. Furthermore, these risk scores exhibited associations with immune checkpoints and chemotherapeutic drug sensitivity. Collectively, our exhaustive analysis of HMRGs in LUAD patients unveiled their conceivable participation in configuring the multifaceted tumor microenvironment, encompassing clinicopathological attributes and prognosis. A sequence of experiments illuminated the pro-proliferative and pro-migratory attributes of KRT8 in vitro and vivo, thus underscoring its carcinogenic potential.

**Conclusions:** In this study, we have unearthed innovative gene signatures tethered to HMRGs, which harbor prognostic implications concerning patient outcomes. These insights hold potential for steering the development of targeted therapeutic modalities tailored for LUAD.

## Introduction

Lung adenocarcinoma (LUAD), a predominant subtype of non-small cell lung cancer, presents a substantial global health challenge, responsible for a significant share of cancer-related fatalities 1, 2. Despite advancements in diagnostic and therapeutic modalities, LUAD patients continue to confront an unfavorable prognosis, underscoring the imperative for a deeper comprehension of the underlying molecular mechanisms steering tumor progression and the identification of novel targets for intervention 3, 4. Within the tumor microenvironment, hypoxia, characterized by oxygen insufficiency, stands as a hallmark feature common to solid tumors, including LUAD 5, 6. Hypoxia triggers a myriad of adaptive responses in cancer cells, notably involving the activation of hypoxia-inducible factors (HIFs) which orchestrate the expression of genes governing angiogenesis, metabolism, and cellular viability 7. Hypoxic conditions within the tumor microenvironment correlate with aggressive tumor behavior, therapy resistance, and poor patient outcomes 8. Furthermore, there is mounting evidence underscoring the pivotal role of mitophagy, a selective form of autophagy dedicated to the targeted removal of damaged mitochondria, in cancer progression and therapeutic responsiveness 9, 10. Concurrently, a growing body of research has spotlighted the intricate interplay between hypoxia and mitophagy in LUAD 11. Nonetheless, the precise mechanisms governing this interaction remain elusive, with limited studies exploring the entirety of hypoxia and mitophagy related genes (HMRGs) and their pathway enrichments in LUAD.

The immune microenvironment occupies a central role in tumor development, progression, and therapeutic response 12, 13. Tumor-infiltrating immune cell subsets, such as T cells, B cells, natural killer cells, and myeloid cells, engage in dynamic interactions with cancer cells within the tumor microenvironment, significantly influencing the immune response against the tumor 14. The immune landscape in LUAD is characterized by marked heterogeneity, exerting profound effects on disease progression, treatment response, and patient outcomes 15. Unraveling the intricate interplay between hypoxia, mitophagy, and the immune microenvironment is pivotal for the identification of innovative therapeutic strategies aimed at enhancing patient outcomes in LUAD.

In this study, we embarked on a comprehensive bioinformatics analysis of HMRGs in LUAD, striving to unveil their molecular attributes and functional implications. We established a signature capable of predicting overall survival (OS) and employed it to characterize the immune milieu in LUAD. The identification of novel gene signatures linked to HMRGs holds promise as potential prognostic biomarkers and therapeutic targets. Collectively, these findings may contribute substantively to the development of immunotherapeutic strategies and the enhancement of clinical outcomes for individuals grappling with LUAD.

## Materials and Methods

### Data Collection and Processing

Fig. S1 shows a map of the process of the present work. We acquired gene expression data, somatic mutation data, and corresponding clinical information for LUAD from the The Cancer Genome Atlas (TCGA) database. Furthermore, datasets GSE31210 and GSE72094 were retrieved from the Gene Expression Omnibus (GEO) database. To mitigate batch effects, we employed the "Combat" algorithm and merged three cohorts, resulting in a study cohort comprising 1,093 patients. Patients who met the following selection criteria were included: (a) histologically diagnosed with LUAD; (b) available gene expression data; (c) available survival and clinical information.

### Generation of HMRGs

We compiled a list of 200 hallmark genes from the Molecular Signatures Database (https://www.gsea-msigdb.org/gsea/msigdb/), categorizing them as hypoxia-related genes. Supplementary Table S1 contains the supplemented list. In addition, we gathered 29 mitophagy-related genes from prior research and literature in the Supplementary Table S2. Our analysis encompassed a total of 229 HMRGs across all cohorts.

### Consensus Clustering Analysis of HMRGs

Utilizing the "ConsensusClusterPlus" package in R 16, we applied unsupervised clustering analysis to categorize patients into distinct molecular subtypes, based on the mRNA expression profiles of HMRGs. Consensus clustering is a conventional method for subtype classification in cancer research. Subtypes are identified through the integration of different omics data sets, enabling the discovery and comparison of disease subtypes. Subtype distribution was verified via principal component analysis utilizing gene expression profiles.

### Differentially Expressed Genes Identification and Functional Enrichment Analysis

Differentially expressed genes (DEGs) were identified using the "limma" package in R between different subtypes 17, employing a fold-change threshold of two and an adjusted p-value threshold of <0.01. Subsequent gene set variation analyses (GSVA) of the DEGs were conducted using the "cluster profile" package in R, aiming to elucidate potential functions and enrichment pathways associated with different HMRGs patterns.

### Construction of the HMRGs Prognostic Signature

Univariate Cox regression analysis was performed to select genes with prognostic significance, considering p-values <0.05 as statistically significant. We randomly partitioned the LUAD patient cohort into training (n = 553) and test (n = 553) sets at a 1:1 ratio. The training set was utilized to formulate the HMRGs prognostic signature. Key genes and their corresponding coefficients for model construction were identified using LASSO Cox regression analysis. Patient risk scores were calculated using the standardized expression levels of these key genes and their corresponding regression coefficients, following this formula: Risk score = ∑ (each gene's expression × corresponding coefficient). The details composition and correlation coefficients of the model genes was supplemented in Table S3. Patients were dichotomized into low- and high-risk groups based on the median risk score. OS analysis among different patient groups with LUAD was conducted employing the "Survival" package.

### Mutation and Drug Susceptibility Analysis

The tumor mutational burden in the TCGA cohort was visualized using the "maftools" package in R. The "pRRophetic" software package was utilized to calculate half-inhibitory concentration (IC50) values for drugs targeting LUAD 18. This analysis aimed to discern disparities in drug sensitivity among patients with varying risk scores.

### Establishment of a Nomogram Scoring System

We employed the "rms" package to construct a nomogram, providing predictive clinical information regarding the clinical attributes and risk scores of LUAD patients, especially regarding 1-, 3-, and 5-year OS. Each clinical variable was assigned a score, with the total score determined by summing across all variables. Calibration plots were employed to assess the predictive accuracy of the nomogram for 1-, 3-, and 5-year OS compared to observed outcomes.

### Assessment of Tumor Microenvironment

The "estimate" package facilitated computation of stromal, immune, and ESTIMATE scores for each sample using the ESTIMATE algorithm. Immune cell infiltration in each sample was evaluated via single-sample Gene Set Enrichment Analysis (ssGSEA).

### Cell Culture and Transfection

Human cancer cell lines A549, HCC827, H1650, PC9, and the normal human bronchial epithelial cell line BEAS-2B were procured from the American Type Culture Collection. Cells were cultured in DMEM with 10% fetal bovine serum and 1% penicillin-streptomycin in a 37°C incubator with 5% CO2. Transfection employed siRNA specifically targeting KRT8 or a negative control siRNA (Ribobio, China) and Lipofectamine 2000 (Invitrogen, USA). The KRT8-targeting siRNA had the sequence: 5′‐CUGAGAUGAACCGGAACAU‐3′.

### Real-time Polymerase Chain Reaction (PCR), Cell Proliferation, EdU Assay, Colony Formation, Transwell Assay, and Scratch Wound-Healing Assay

Detailed protocols for these experiments were executed following standard procedures as described in the previous study 19. The primers used in this study were supplemented in Table S4.

### Animal model

BALB/c mice (female, 4-6 weeks of age, 18-20 g) were housed in a specific pathogen-free (SPF) environment. In total, 2 × 10^4^ cells (A549, A549-sh-KRT8-1, or A549-sh-KRT8-2) were injected subcutaneously into the right flank of the mice. After 24 days, the tumors were surgically dissected. All animal experiments were performed according to the procedures approved by the institutional animal care and use committee of Tianjin Medical University General Hospital.

### Statistical Analysis

All statistical analyses were carried out using R version 4.1.2. Kaplan-Meier analysis was employed to compare overall survival between subgroups. Time-dependent receiver operating characteristic (ROC) curve analysis assessed the predictive value of the risk score, with p < 0.05 indicating statistical significance.

## Results

### Molecular Patterns of HMRGs with Distinct Survival in LUAD

**Figure 1:**
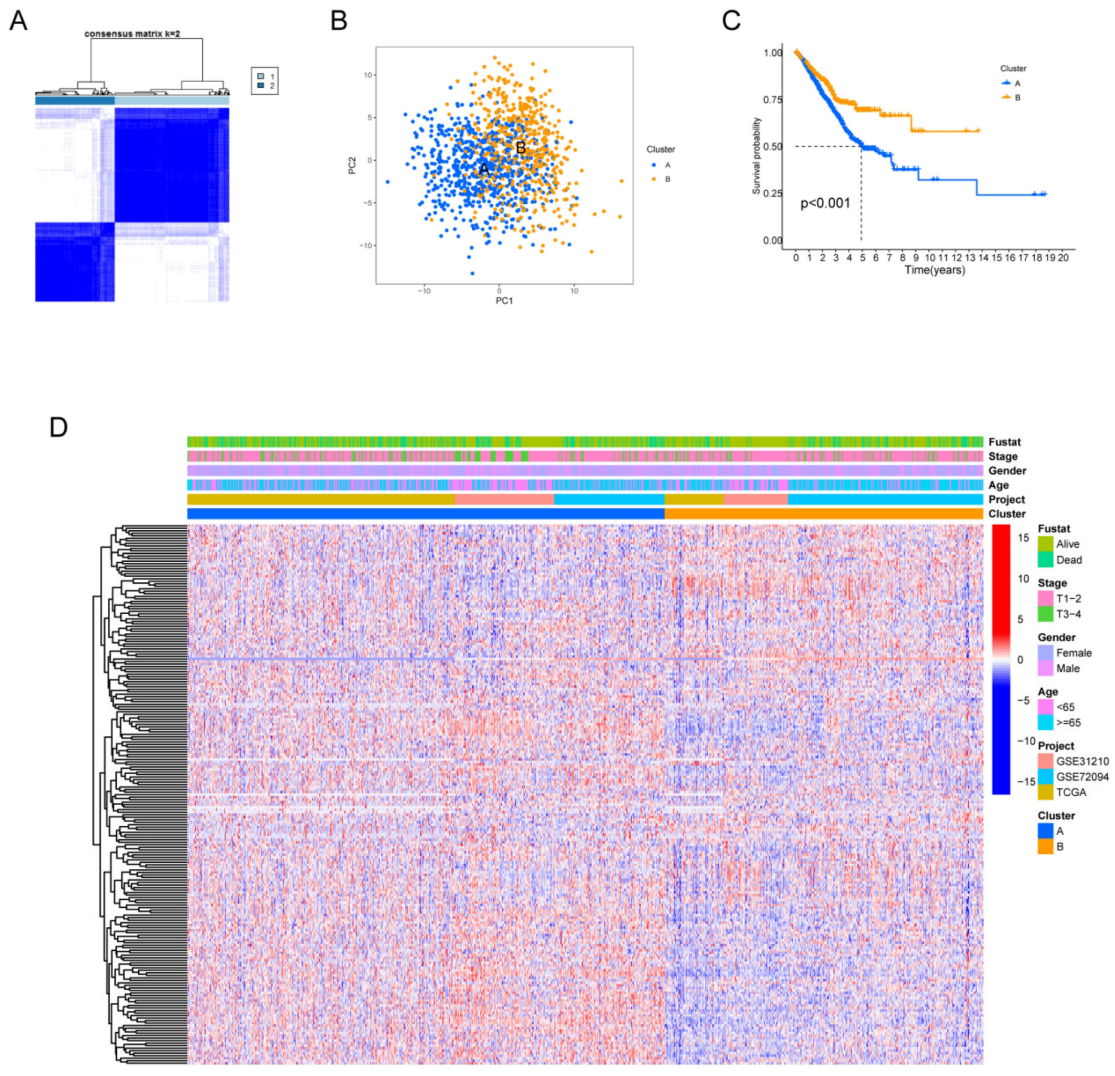
Hypoxia and mitophagy clusters in the LUAD. (A) The consensus matrix heatmap defines two clusters (k = 2) and their correlation area. (B) Principal Component Analysis between the two clusters. (C) Kaplan-Meier curve illustrating the survival differences between the two clusters of LUAD patients. (D) Heatmap displaying the relationships between clinicopathological characteristics of the patients and the two clusters.

Fig. S1 provides a comprehensive overview of our research process. Our study delved deeply into the biological characteristics and expression profiles of HMRGs in LUAD. Employing unsupervised clustering analysis based on the expression profiles of the 229 HMRGs, we stratified LUAD patients into distinct subgroups. The consensus cumulative distribution function curve identified k = 2 as the optimal choice, bifurcating the entire cohort into subtype clusters A and B (Fig. 1A). Principal component analysis (PCA) further underscored conspicuous differences in transcription profiles of HMRGs between these subtypes (Fig. 1B). Notably, patients in cluster A exhibited a significantly shorter OS, as evidenced by Kaplan-Meier curves (Fig. 1C). The distinct clinicopathological features of patients in LUAD subtypes are visually depicted in Fig. 1D.

### Identifying HMRGs Subtypes via Differentially Expressed Genes

**Figure 2:**
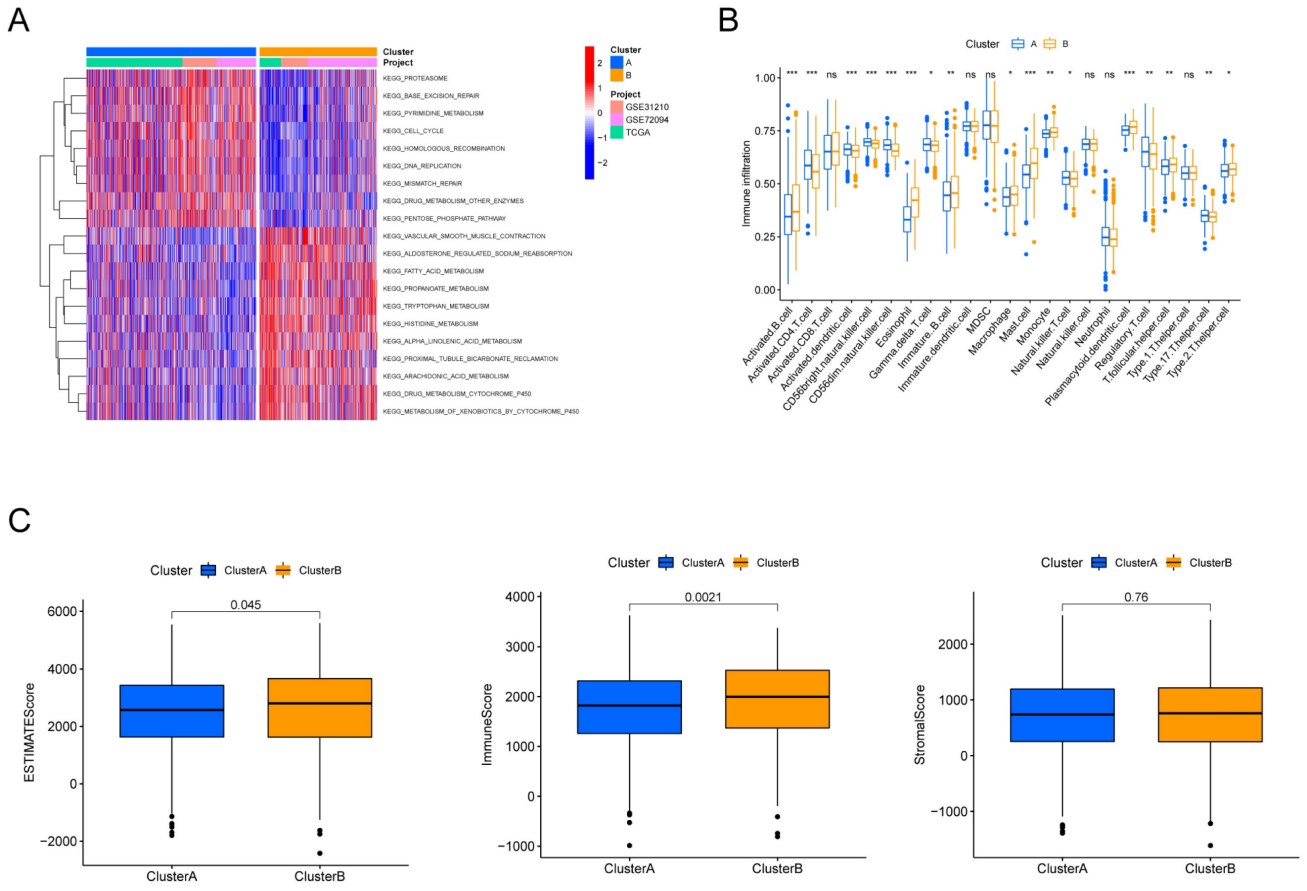
Discrepancy in immune infiltration between the two clusters. (A) Gene set variation pathways between the two clusters. (B) Comparison of immune cell fractions between the two clusters using the CIBERSORT method. (C) Differences in ESTIMATE scores, immune scores and stromal scores between the two clusters.

To explore the potential biological behaviors of the HMRGs subtypes, we identified HMRGs subtype-related DEGs and conducted functional enrichment analysis using the R package "limma". GSVA revealed significant enrichment of these DEGs in specific biological processes, including proteasome function, base excision repair, and pyrimidine metabolism (Fig. 2A). Additionally, we delved into the relationship between these clusters and the characteristics of the tumor microenvironment (TME). Notably, cluster A exhibited significantly higher scores for activated CD4 T cells, activated dendritic cells, CD56dim natural killer cells, gamma delta T cells, natural killer T cells, Regulatory T cells, and type 17 T helper cells (Fig. 2B). Furthermore, patients in cluster A displayed distinctly lower estimate scores and immune scores, although stromal scores remained relatively uniform (Fig. 2C).

**Figure 3:**
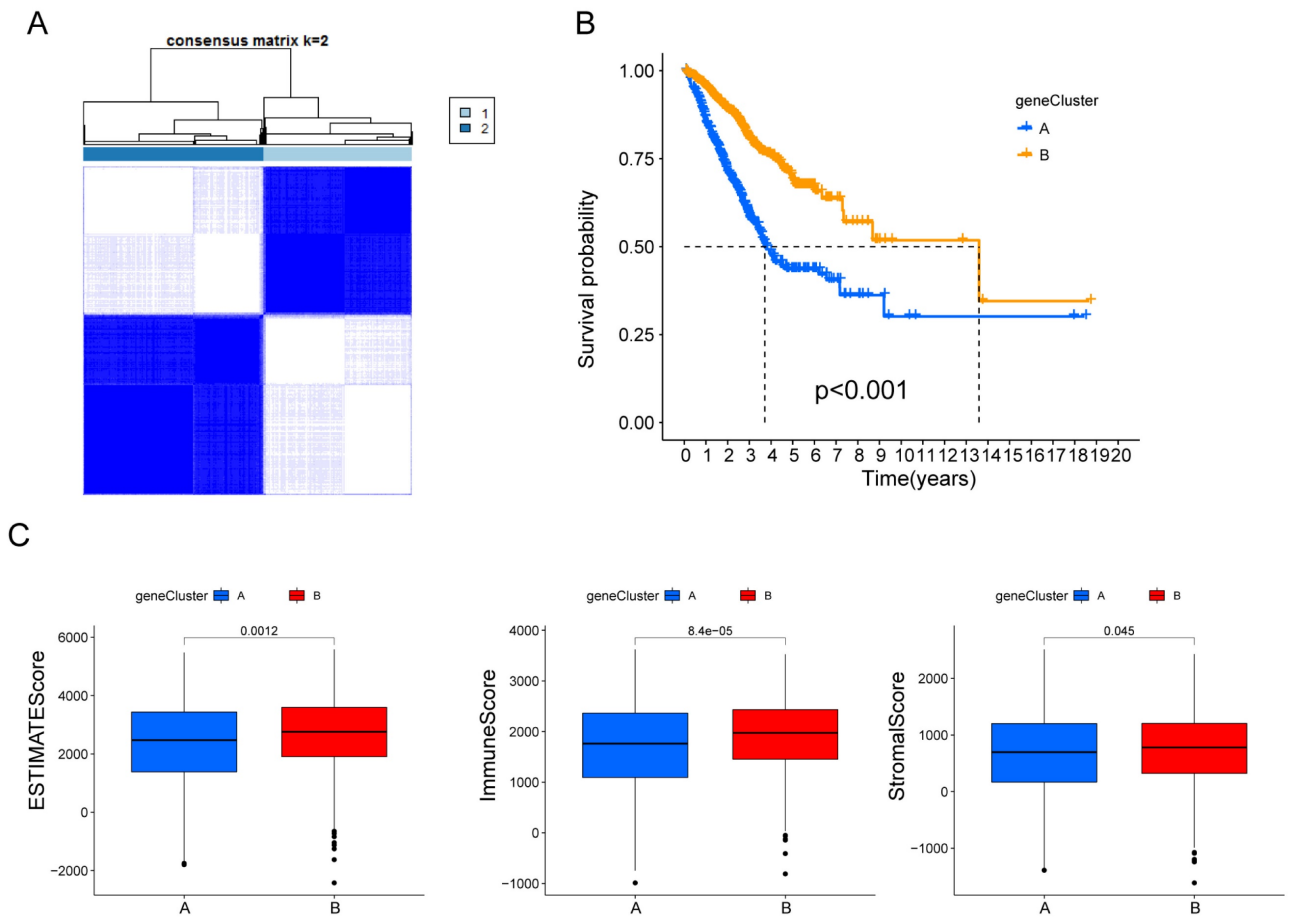
Identification of hypoxia and mitophagy gene clusters based on differentially expressed genes. (A) The consensus matrix heatmap defines the two gene clusters (k = 2) and their correlation area. (B) Kaplan-Meier curves for the two gene clusters (log-rank tests, p < 0.001). (F) Differences in ESTIMATE scores, immune scores and stromal scores between the two gene clusters.

Subsequently, the patients were divided into two genomic subtypes based on prognostic genes using an unsupervised clustering analysis to further investigate the special regulation mechanism (Fig. 3A). The OS time of the patients in the gene cluster A was worse than those in the gene cluster B per the results of Kaplan-Meier curves (Fig. 3B). Furthermore, patients in gene cluster A displayed distinctly lower estimate scores, immune scores and stromal scores (Fig. 3C).

### Development and Validation of HMRGs Signature

**Figure 4:**
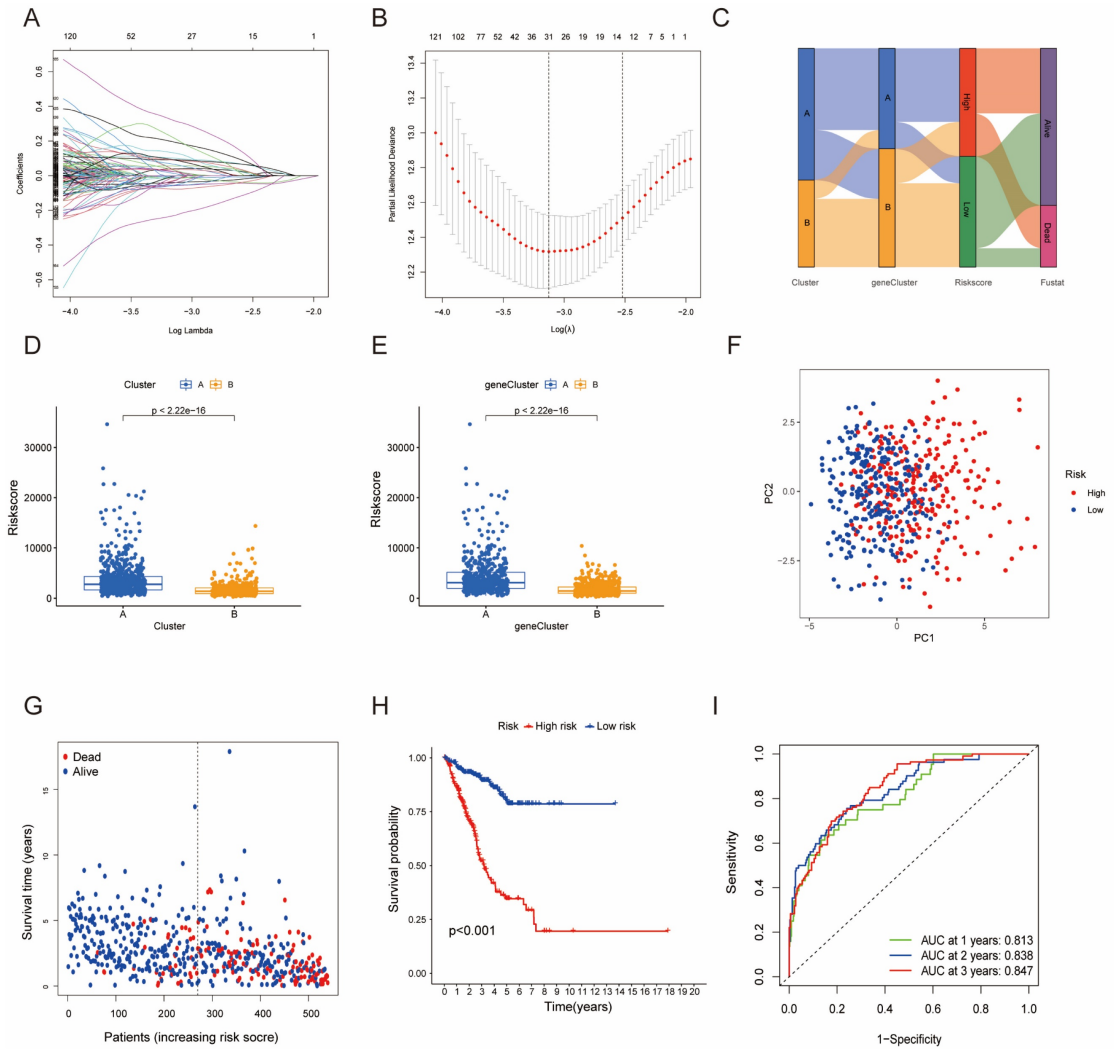
Construction of a predictive model of LUAD in the training cohort. (A, B) Selection of the optimal parameter (lambda), represented by the vertical black line in the plot. (C) Alluvial diagram depicting the relationship between the cluster, gene cluster, risk score, and survival outcome group. (D) Boxplot of the risk scores between the clusters. (E) Boxplot of the risk scores between the two gene clusters. (F) PCA analysis illustrating significant differences between high-risk and low-risk patients. (G, H) Distributions of OS status and OS of patients between high-risk and low-risk groups, with higher score values and mortality in the high-risk group. (I) Time-independent ROC analysis of the risk score for predicting OS, with the area under the curve for 1, 2, and 3 years reaching 0.813, 0.838, and 0.847, respectively.

**Figure 5:**
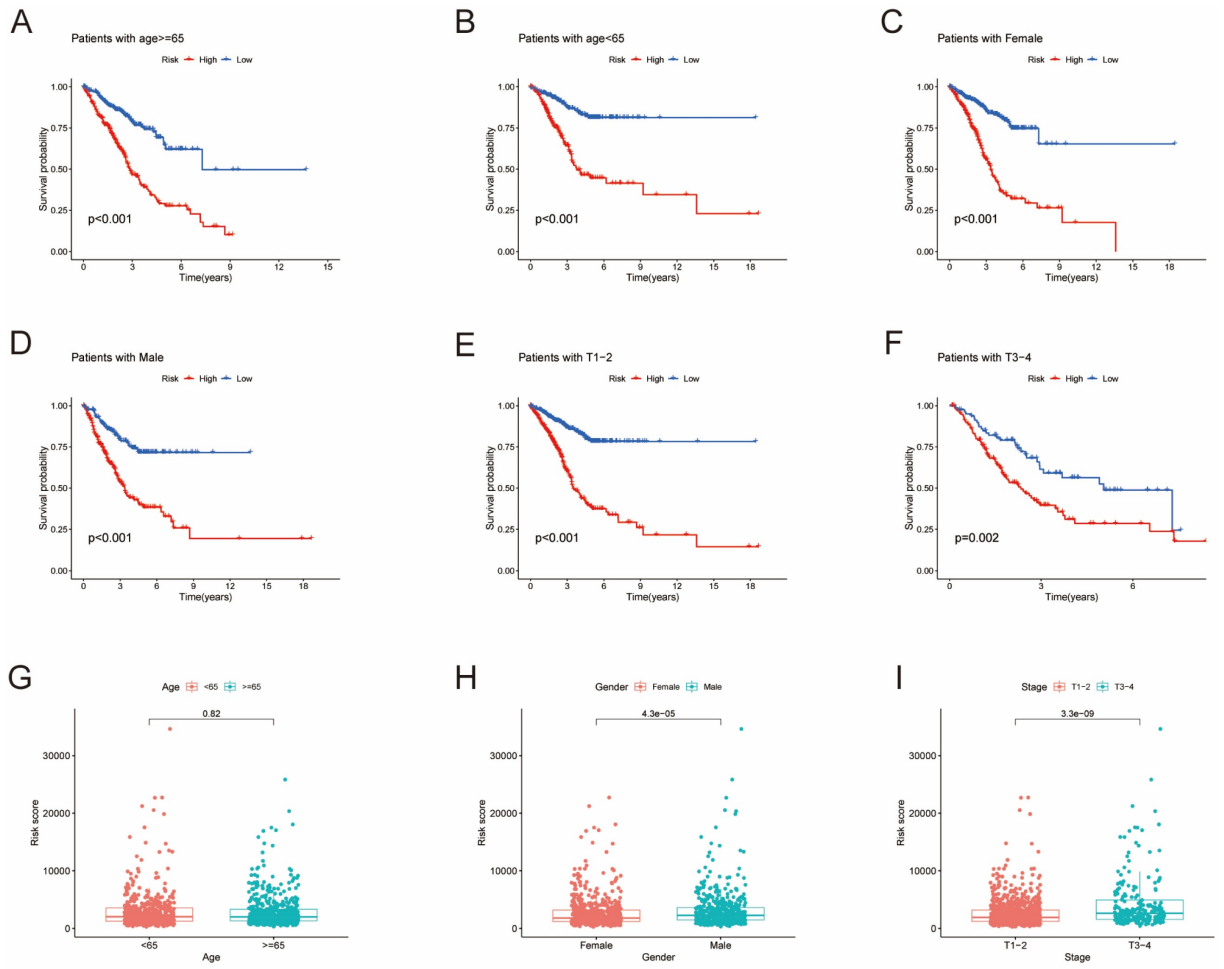
Survival analysis of clinical stratification of OS in the training cohort. (A, B) Age stratification (< 65 or ≥ 65 years old). (C, D) Gender stratification (female or male). (E, F) Tumor stage (T1-2 or T3-4). (G) Boxplot of the risk scores between age groups (< 65 or ≥ 65 years old). (H) Boxplot of the risk scores between gender groups (female or male). (I) Boxplot of the risk scores between tumor stage groups (T1-2 or T3-4).

**Figure 6:**
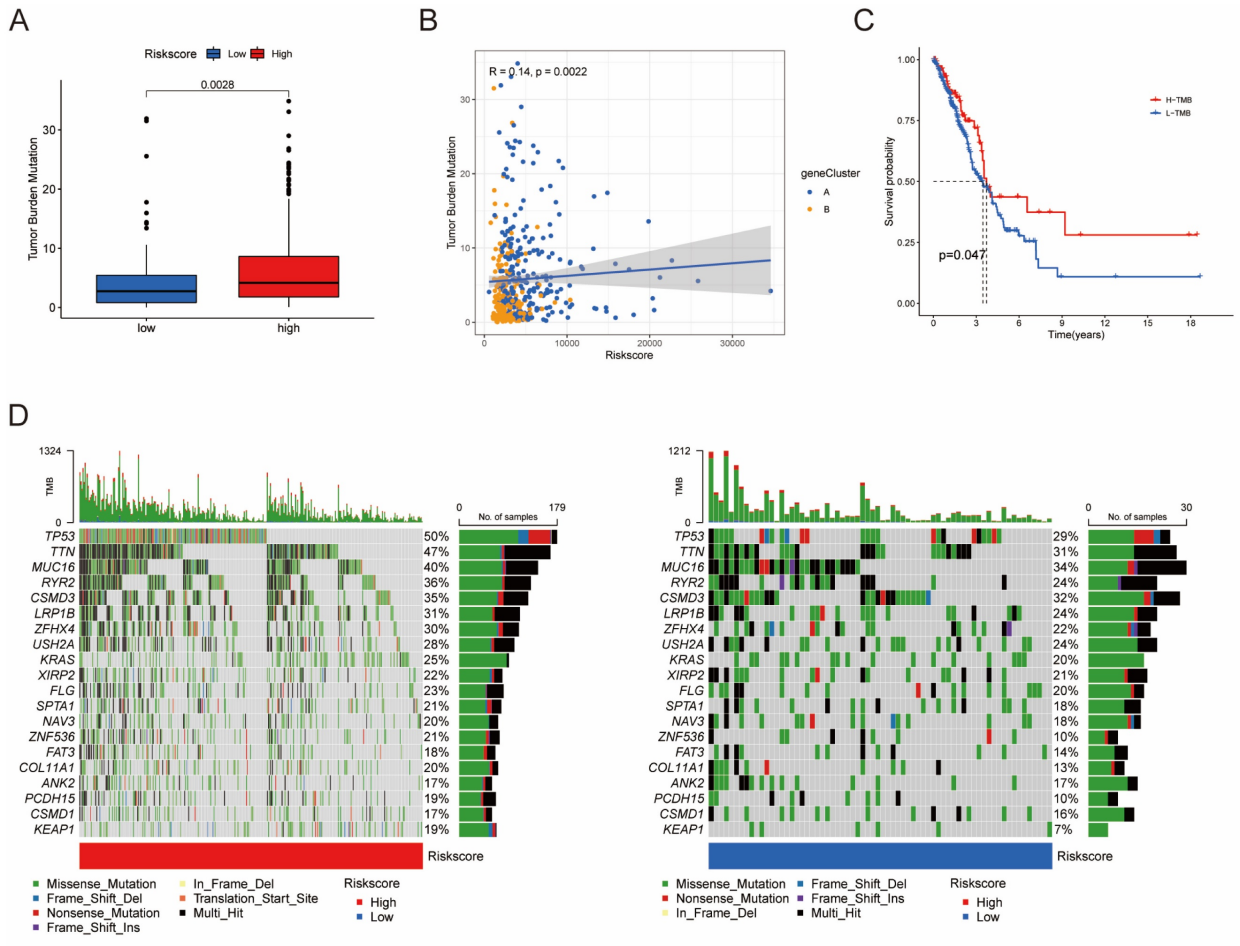
Differences in tumor mutational burden (TMB) between high and low risk score groups. (A) Boxplot illustrating TMB differences between the groups. (B) Correlation analysis between risk scores and TMB. (C) Survival analysis of OS between the TMB subgroups. (D) Top 20 mutated genes shown between the high- and low-risk groups.

Furthermore, univariate Cox regression analysis was performed to identify the genes possessing prognostic values. We randomly partitioned patients into training and validation cohorts to construct a prognostic signature using LASSO Cox regression analysis based on the gene with prognostic values (Fig. 4A, B). This signature facilitated the calculation of risk scores for each LUAD patient. Subsequently, patients were divided into high and low risk score groups (Fig. 4C). Notably, cluster A exhibited the higher risk scores compared to cluster B (Fig. 4D). The same trend was observed within gene clusters, where gene cluster A also exhibited the higher risk scores (Fig. 4E). PCA analysis demonstrated distinctive patient clustering based on the median risk score (Fig. 4F). Furthermore, patients in the high risk score group experienced poorer OS within the training cohort (Fig. 4G, H). The ROC curve affirmed the strong prognostic value of our model (Fig. 4I), a trend consistent across the test and entire datasets (Fig. S2A-H). To validate the prognostic reliability across various clinical subgroups, we conducted a comprehensive analysis. High score patients demonstrated poorer prognoses within age, gender, and T grade subgroups (Fig. 5A-F). Additionally, we noted differences in risk scores across these subgroups, especially in males and stage T3-4 patients, who exhibited significantly higher risk scores (Fig. 5G-I). Genomic mutation comparisons between high- and low-risk groups revealed higher tumor mutational burden (TMB) in the high-risk group, with a positive correlation between risk score and TMB score (Fig. 6A, B). Moreover, low TMB patients exhibited reduced OS (Fig. 6C), and mutational profiles also varied between high- and low-risk groups (Fig. 6D).

### Building a Prognostic Nomogram and Analyzing Drug Susceptibility

**Figure 7:**
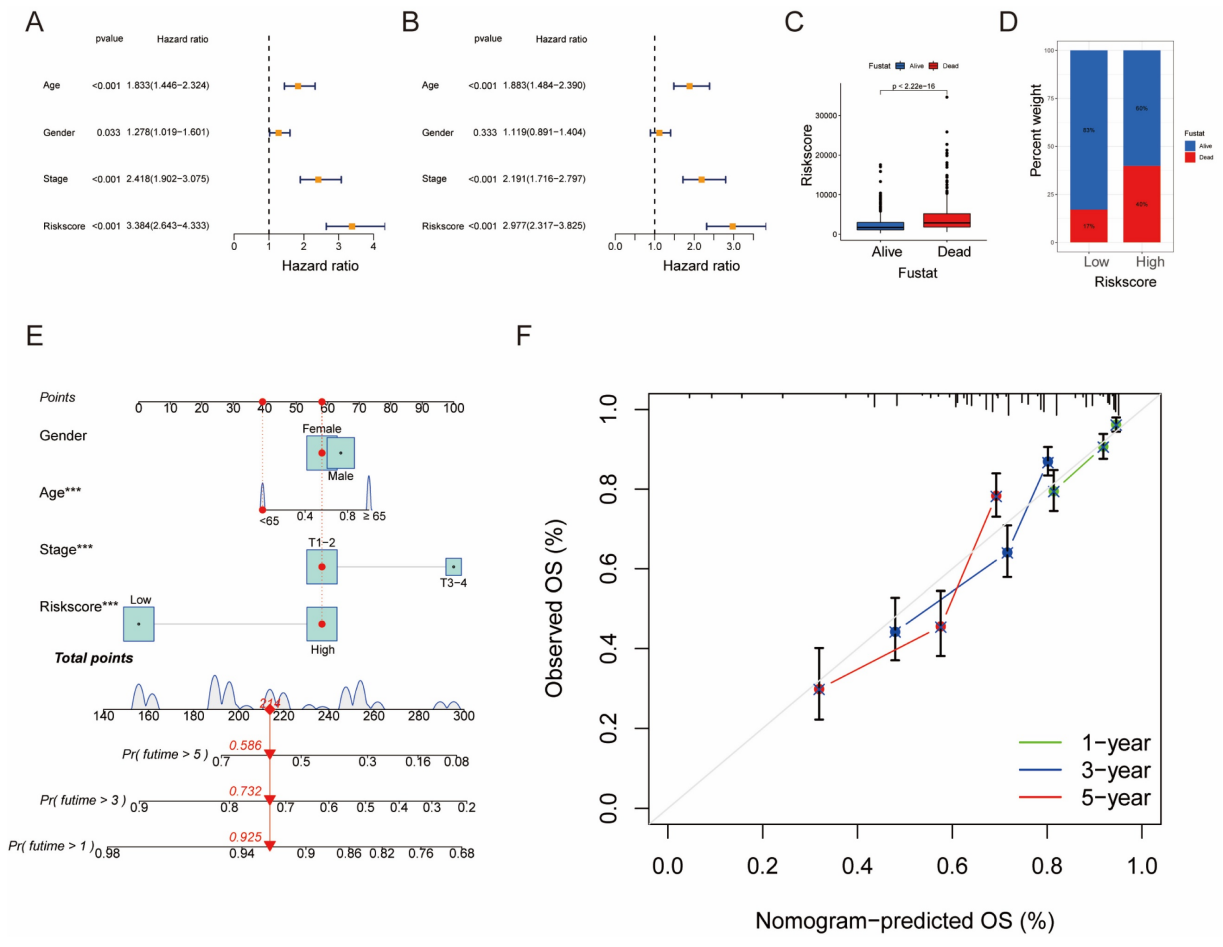
Establishment and Confirmation of a Nomogram. (A, B) Forest plot displaying the results of univariate and multivariate Cox regression analyses regarding OS in the entire cohort. (C) Boxplot of the risk scores based on the OS status of patients. (D) Proportion of patients with vital status in the high-risk and low-risk groups. (E) Nomogram for predicting the 1-, 3-, and 5-year OS of LUAD patients in the training set. (F) Calibration curves of the nomogram for predicting 1-, 3-, and 5-year OS in the training set.

Univariate and multivariate Cox analyses demonstrated the prognostic significance of risk scores for OS (Fig. 7A, B). Notably, patients with higher risk scores exhibited higher mortality rates (Fig. 7C, D). A novel nomogram OS prediction model was developed, integrating risk scores with clinicopathological parameters to enhance predictive accuracy (Fig. 7E). The calibration curve affirmed the high accuracy of this nomogram in predicting LUAD outcomes (Fig. 7F).

### Assessing Immune Infiltration and Checkpoints

**Figure 8:**
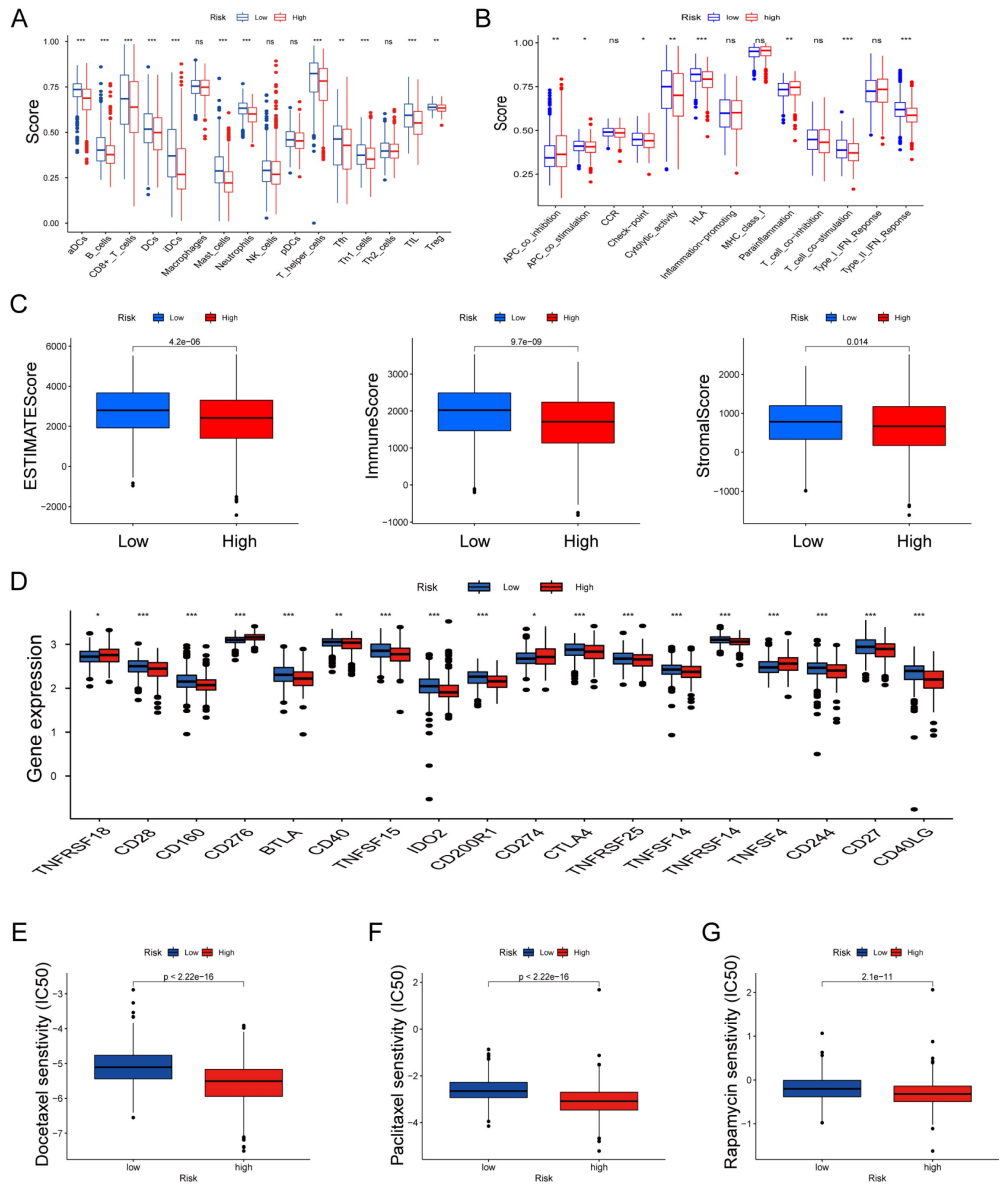
Features of the tumor microenvironment in the high and low risk score groups of LUAD. (A) Abundance of 16 infiltrating immune cell types in the high and low risk score groups. (B) Correlation of risk scores with 13 immune functions. (C) Differences in immune scores, ESTIMATE scores, and stromal scores between the different risk score groups. (D) Differential expression of common immune checkpoints between the different risk score groups. Boxplots depict differences in estimated IC50 levels of (E) docetaxel, (F) paclitaxel, and (G) rapamycin between risk score and chemotherapeutic sensitivity; ^ns^p ≥ 0.05, *p < 0.05, **p < 0.01, ***p < 0.001.

We further explored the relationship between risk scores and TME characteristics. Patients in the low risk score group displayed significantly higher scores for various immune cells and immune-related functions (Fig. 8A, B). In contrast, patients in the high risk score group exhibited lower estimate scores, immune scores, and stromal scores (Fig. 8C). We also scrutinized the association between immune checkpoints and our risk model, revealing distinct immune checkpoint expressions between the two risk groups, including CD28, CD160, BTLA, CD40, CD274 and CTLA4. et al (Fig. 8D). Furthermore, we calculated the IC50 values of commonly used chemotherapeutic drugs for LUAD treatment using the "pRRophetic" package, noting that patients with high risk scores exhibited lower IC50 values for docetaxel, paclitaxel, and rapamycin (Fig. 8E-G).

### KRT8 Promotes Proliferation and Migration of Lung Cancer

**Figure 9:**
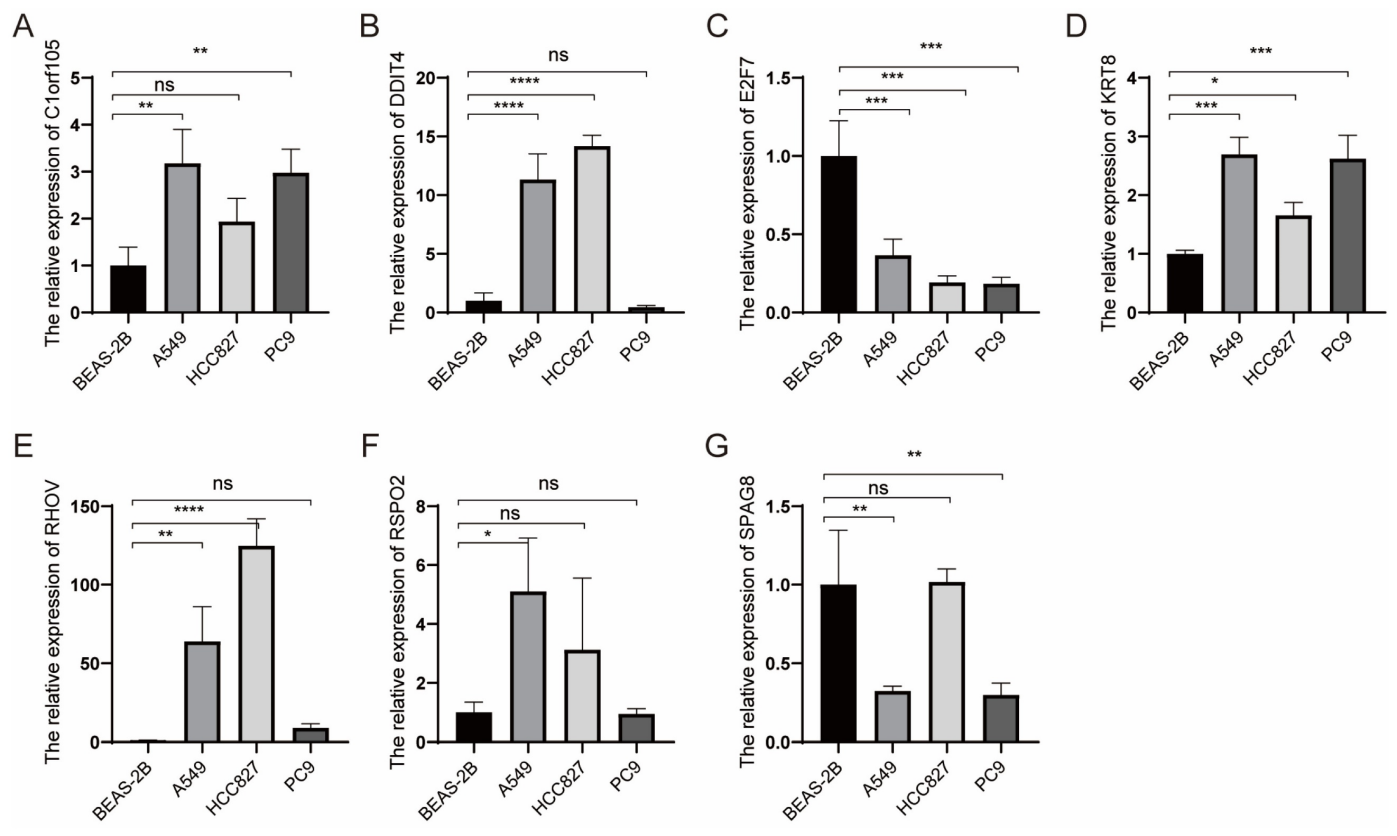
Relative expression of selected genes in various cell lines. (A-G) Relative expression levels of Clorf105, DDIT4, E2F7, KRT8, RHOV, RSPO2, and SPAG8 measured by qRT-PCR in BEAS-2B, A549, HCC827, and PC9 cells. ^ns^p ≥ 0.05, *p < 0.05, **p < 0.01, ***p < 0.001, ****p < 0.0001, compared with the control group.

**Figure 10:**
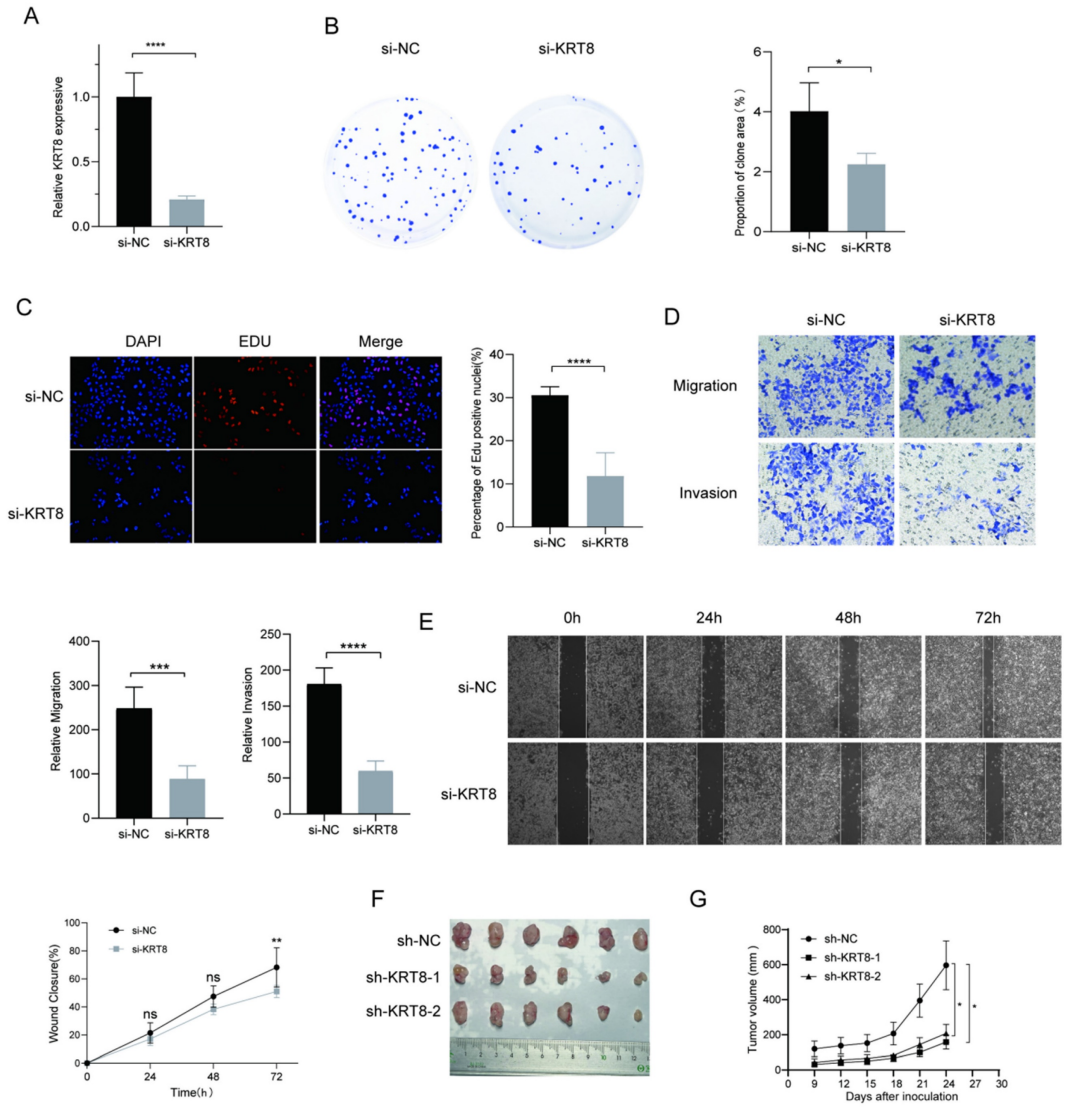
Functional Analysis of KRT8 in A549 Cells. (A) Efficiency of KRT8 knockdown in A549 cells measured by qRT-PCR. (B) Inhibition of colony-forming capacity in KRT8 knockdown A549 cells as assessed by a colony-formation assay. (C) Evaluation of cell proliferation in control and KRT8 knockdown A549 cells using EdU staining. (D) Inhibition of A549 cell invasion in KRT8 knockdown A549 cells as measured by a transwell assay. (E) Confirmation of impaired cell wound-healing ability in KRT8 knockdown cells using wound-healing experiments. (F, G) A549, A549-sh-KRT8-1, or A549-sh-KRT8-2 were injected were injected into BALB/c mice, photograph of dissected tumors (n = 6). Error bars represent the mean ± SD, and data are from three independent experiments. Two-sided t tests were applied for statistical analysis. *P < 0.05, **P < 0.01, ***P < 0.001, ****P < 0.0001, compared with the control group.

To further elucidate genes influencing LUAD malignancy, we performed DEGs analysis of signature genes in LUAD and paracancerous tissues. Afterwards, we assessed the relative expression of Clorf105, DDIT4, E2F7, KRT8, RHOV, RSPO2, and SPAG8 in BEAS-2B, A549, HCC827, and PC9 cells via qRT-PCR. The results consistently showed significant overexpression of KRT8 in all lung tumor cell lines (Fig. 9). We validated the efficiency of siRNAs targeting KRT8 via qRT-PCR and evaluated their impact on cell proliferation, migration, and invasion. KRT8 knockdown significantly inhibited proliferation, migration, and invasion of A549 cells compared to the control group (Fig. 10A-D). Moreover, in the cell scratch assay, KRT8 knockdown significantly impaired cell migration compared to the control group, demonstrating its role in promoting lung cancer cell migration (Fig. 10E). To further verify the effect of KRT8 observed in vitro, we subcutaneously injected A549 cells and KRT8-knockdown A549 cells into BALB/c mice. Knockdown of KRT8 appreciably attenuated tumor growth in the mice (Fig. 10 F, G).

## Discussion

The main purpose of this study is the study of hypoxia and mitophagy related gene signature in LUAD prognostic role. while also aiming to characterize the tumor immune microenvironment. These findings illuminate the potential roles of these molecular pathways in tumor progression and mechanisms of immune evasion.

An analysis of hypoxia-related genes uncovered a substantial upregulation of HIF-1α, HIF-2α, and VEGF in LUAD samples compared to normal lung tissue 20. HIFs play pivotal roles in cellular adaptation to hypoxic conditions and have been associated with various aspects of tumor biology, including angiogenesis, metastasis, and resistance to treatment 21. The overexpression of HIF-1α and HIF-2α in LUAD suggests the activation of hypoxia-responsive signaling pathways, potentially contributing to tumor aggressiveness and adversely affecting patient prognosis 22. Survival analysis corroborated these findings, underscoring the prognostic relevance of high expression levels of HIF-1α, HIF-2α, and VEGF. Additionally, an examination of mitophagy-related genes identified an upregulation of PINK1, Parkin, and LC3 in LUAD samples. Mitophagy, a selective form of autophagy, is responsible for eliminating damaged mitochondria and preserving cellular homeostasis 23. Dysregulation of mitophagy has been implicated in the development and progression of cancer. The elevated expression of mitophagy-related genes in our study suggests a potential role for mitophagy dysregulation in the pathogenesis of LUAD. Notably, our correlation analysis unveiled an association between hypoxia and mitophagy-related genes signature and immune checkpoint molecules, suggesting a potential interplay between hypoxia and mitophagy dysregulation and mechanisms of immune evasion in LUAD.

In characterizing the immune microenvironment, we observed heterogeneous patterns of immune cell infiltration and differential expression of immune checkpoint genes within different score subtypes. Tumor-infiltrating lymphocytes, including CD8+ T cells, CD4+ T cells, B cells, NK cells, and myeloid cells, displayed varying levels of infiltration, indicating dynamic interactions between our signature and immune cell infiltration. The differential expression of immune checkpoint molecules, such as PD-L1 and CTLA-4, suggested potential immune evasion strategies employed by the tumor to evade immune surveillance. Analyzing the differences in various molecular features within the immune microenvironment contributes to accurately predicting patient prognosis and lays the foundation for developing personalized immunotherapy strategies.

The KRT8 gene, encoding a protein known as Keratin 8, plays a significant role in the realm of lung cancer diagnosis and treatment. Keratin 8 is a structural protein found predominantly in various epithelial cells 24, 25. Its primary function lies in providing cellular structural support and maintaining mechanical stability. Our study suggests that the expression of KRT8 may be associated with the aggressiveness and prognosis of LUAD. In the future, targeting the expression level of KRT8 may be a potential therapeutic target for LUAD, with individualized treatment tailored to the needs of each LUAD patient.

In conclusion, this study offers valuable insights for future clinical practices, potentially enhancing methods for disease diagnosis, treatment, or prevention. These results could serve as a foundation for the development of new clinical strategies, drug formulations, or personalized treatment plans. Furthermore, our research outcomes may impact the guiding principles of medical practices, providing more precise information for clinical decision-making and ultimately improving the therapeutic outcomes and quality of life for patients. The identification of prognostic biomarkers and potential therapeutic targets within these pathways holds promise for personalized treatment strategies in LUAD.

## Supplementary Material

Supplementary figures and tables.
