# Supplementary material for: Genomic analysis of hypoxia and mitophagy related genes with prognosis and characterization of the immune microenvironment in LUAD
Source: J Cancer. 2024 Jan 16;15(5):1342–54. doi: 10.7150/jca.91762 (PMC10861832; doi:10.7150/jca.91762)
Supplement: Supplementary file 1 — Supplementary figures and tables. [file jcav15p1342s1.pdf]

Figure S1. The entire analytical process of the study.

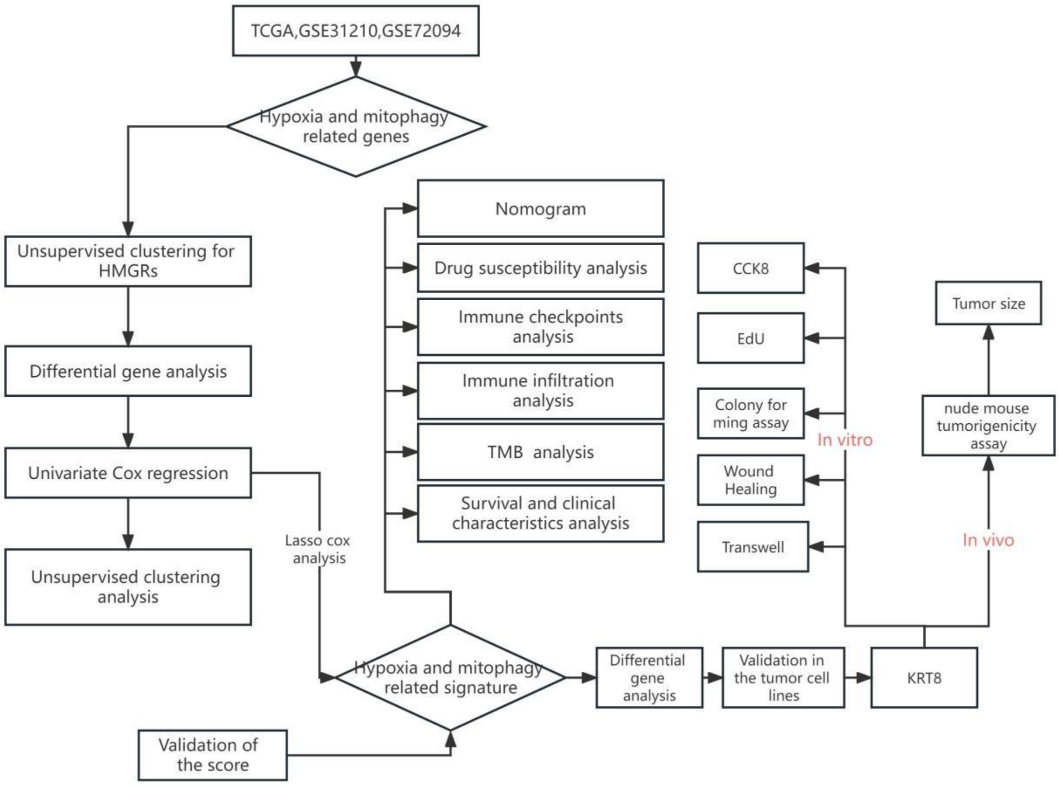

Figure S2

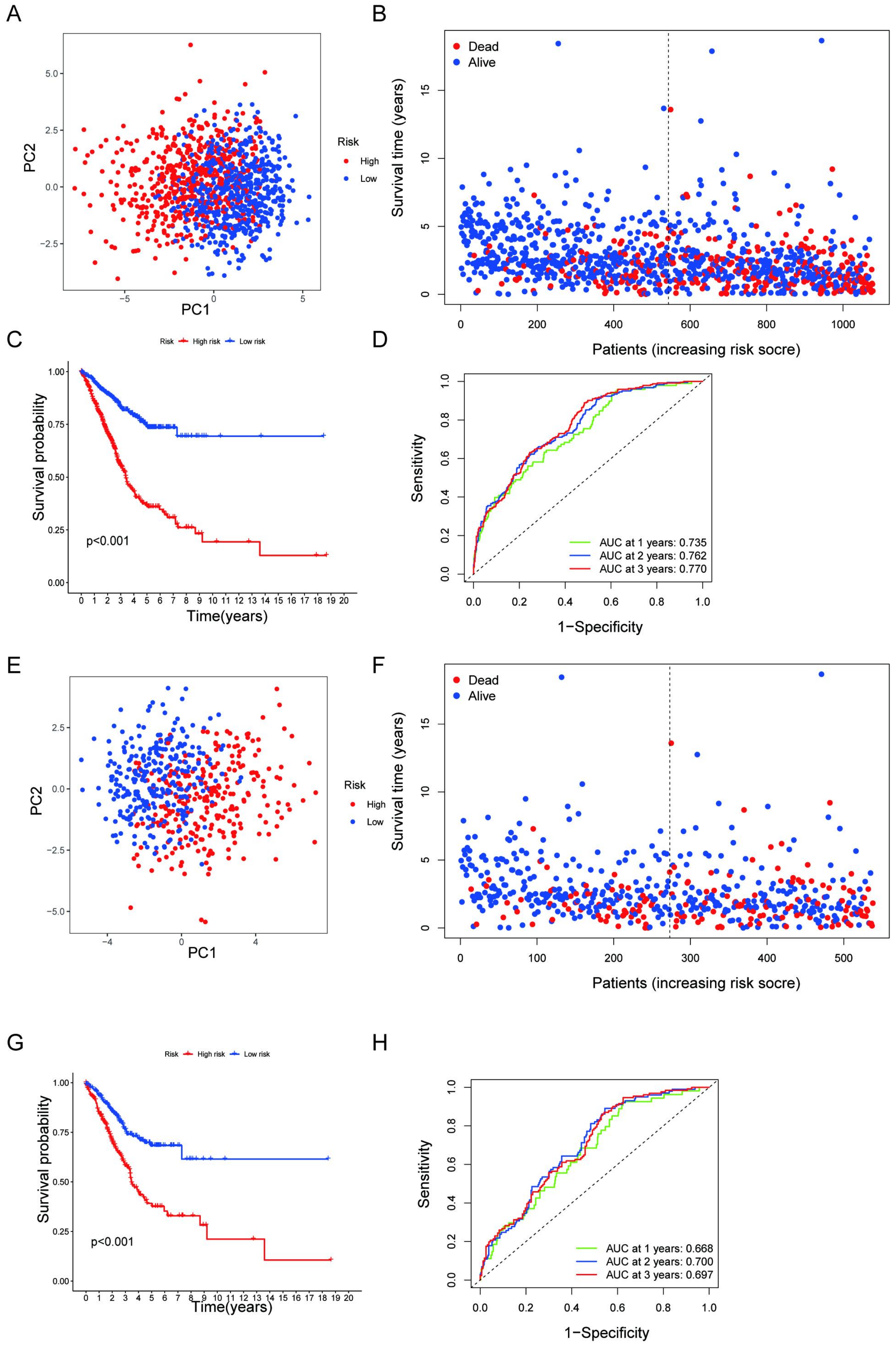

Figure S2: (A) PCA analysis demonstrating significant differences between high-risk and low-risk patients in the test cohort. (B, C) Distribution of OS status and OS of

patients between high-risk and low-risk groups, with patients in the high-risk group showing higher score values and mortality in the test cohort. (D) Time-independent ROC analysis of the risk score for predicting OS, with the area under the curve for 1, 2, and 3 years reaching 0.735, 0.762, and 0.770, respectively, in the test cohort. (E) PCA analysis showing significant differences between high-risk and low-risk patients in the entire cohort. (F, G) Distribution of OS status and OS of patients between high-risk and low-risk groups, with patients in the high-risk group having higher score values and mortality in the entire cohort. (H) Time-independent ROC analysis of the risk score for predicting OS, with the area under the curve for 1, 2, and 3 years reaching 0.668, 0.700, and 0.697, respectively, in the entire cohort.

Table S1. Summary of hypoxia-related genes.

Hypoxia-related genes				
ACKR3	CSRP2	HDLBP	NDRG1	S100A4
ADM	CXCR4	HEXA	NDST1	SAP30
ADORA2B	DCN	HK1	NDST2	SCARB1
AK4	DDIT3	HK2	NEDD4L	SDC2
AKAP12	DDIT4	HMOX1	NFIL3	SDC3
ALDOA	DPYSL4	HOXB9	NOCT	SDC4
ALDOB	DTNA	HS3ST1	NR3C1	SELENBP1
ALDOC	DUSP1	HSPA5	P4HA1	SERPINE1
AMPD3	EDN2	IDS	P4HA2	SIAH2
ANGPTL4	EFNA1	IER3	PAM	SLC25A1
ANKZF1	EFNA3	IGFBP1	PCK1	SLC2A1
ANXA2	EGFR	IGFBP3	PDGFB	SLC2A3
ATF3	ENO1	IL6	PDK1	SLC2A5
ATP7A	ENO2	ILVBL	PDK3	SLC37A4
B3GALT6	ENO3	INHA	PFKFB3	SLC6A6
B4GALNT2	ERO1A	IRS2	PFKL	SRPX
BCAN	ERRFI1	ISG20	PFKP	STBD1
BCL2	ETS1	JMJD6	PGAM2	STC1
BGN	EXT1	JUN	PGF	STC2
BHLHE40	F3	KDELR3	PGK1	SULT2B1
BNIP3L	FAM162A	KDM3A	PGM1	TES
BRS3	FBP1	KIF5A	PGM2	TGFB3
BTG1	FOS	KLF6	PHKG1	TGFBI
CA12	FOSL2	KLF7	PIM1	TGM2
CASP6	FOXO3	KLHL24	PKLR	TIPARP
CAV1	GAA	LALBA	PKP1	TKTL1

CAVIN1	GALK1	LARGE1	PLAC8	TMEM45A
CAVIN3	GAPDH	LDHA	PLAUR	TNFAIP3
CCN1	GAPDHS	LDHC	PLIN2	TPBG
CCN2	GBE1	LOX	PNRC1	TPD52
CCN5	GCK	LXN	PPARGC1A	TPI1
CCNG2	GCNT2	MAFF	PPFIA4	TPST2
CDKN1A	GLRX	MAP3K1	PPP1R15A	UGP2
CDKN1B	GPC1	MIF	PPP1R3C	VEGFA
CDKN1C	GPC3	MT1E	PRDX5	VHL
CHST2	GPC4	MT2A	PRKCA	VLDLR
CHST3	GPI	MXI1	PYGM	WSB1
CITED2	GRHPR	MYH9	RBPJ	XPNPEP1
COL5A1	GYS1	NAGK	RORA	ZFP36
CP	HAS1	NCAN	RRAGD	ZNF292

Table S2. Summary of mitophagy-related genes.

mitophagy-related genes				
ATG12	MAP1LC3A	PINK1	TOMM22	UBA52
ATG5	MAP1LC3B	PRKN	TOMM40	UBB
CSNK2A1	MFN1	RPS27A	TOMM5	UBC
CSNK2A2	MFN2	SQSTM1	TOMM6	ULK1
CSNK2B	MTERF3	SRC	TOMM7	VDAC1
FUNDC1	PGAM5	TOMM20	TOMM70	

Table S3. Composition and correlation coefficients of the model genes.

Gene	Coef
RSPO2	0.024492365
SCARNA17	-0.056782396
IGLC1	-0.03907669
E2F7	0.039422522
ATP8A1	-0.012155166
PMEPA1	0.021919653
RRM1	0.025439152
KRT8	0.055842854
RHOV	0.100350605
DDIT4	0.028346796
AMT	-0.077985661
ABCD1	0.062979162
LINC00324	-0.038423804
CD99	0.190942904

IL27RA	-0.023125091
TXNDC11	-0.014783862
PSMB7	0.128662048
ANO1	0.010028674
C1orf105	0.021486414
MGAT5B	0.037014019
RSAD1	-0.026144302
IGFBP1	0.091480139
FJX1	0.088488225
SPAG8	-0.001316037
SARM1	-0.22639413
BCAP31	0.209184934
GALT	-0.051968202
ASCC1	0.312490847
TBC1D10C	-0.054093371
IKZF3	-0.002042239
CSNK1E	0.08868312

Table S4. Primers used in this study.

Gene	Forward primer	Reverse primer
RSPO2	GACGCAGTAAGCGAGCTAGTT	ACATCGGCTACACCCATTGTC
E2F7	AGGCAGCCCAGACTAGATTTT	GCTGGCAGCACTAATGAGCA
KRT8	CAGAAGTCCTACAAGGTGTCCA	CTCTGGTTGACCGTAACTGCG
RHOV	CCTCATCGTCAGCTACACCTG	GAACGAAGTCGGTCAAAATCCT
DDIT4	TGAGGATGAACACTTGTGTGC	CCAACTGGCTAGGCATCAGC
C1orf105	ATTCACTACAGACTGCCCATTCT	CGTTGTCTTGCCTATTGGTTCC
SPAG8	AGACCAACGAGTCTACGGAGG	GACCTGGGACTGTCATCTGAA
